# Properties of Gangue Powder Modified Fly Ash-Based Geopolymer

**DOI:** 10.3390/ma16165719

**Published:** 2023-08-21

**Authors:** Tianhao Zhang, Zhenghui Yang, Dongsheng Zhang, Qiuning Yang

**Affiliations:** 1 School of Civil and Hydraulic Engineering, Ningxia University, Yinchuan 750021, China; a15304509649@163.com (T.Z.); yzh199802@163.com (Z.Y.); 2 Research Group RecyCon, Department of Civil Engineering, Katholieke Universiteit Leuven, Campus Bruges, 8200 Bruges, Belgium; dongsheng.zhang@kuleuven.be

**Keywords:** geopolymer, gangue powder, mechanical properties, drying shrinkage, microstructure

## Abstract

The environmental and economic problems caused by gangue accumulation continue to worsen. Therefore, the implementation of a cost-effective method for utilizing gangue resources is urgent. In this study, different gangue powder (GP) contents (0%, 10%, 20%, 30%, 40%, and 50%) for mechanical–thermal activation were used to modify a fly ash-based geopolymer (FAG). Further, the effect of GP was revealed by investigating the setting time, fluidity, porosity, water absorption rate, mechanical properties, drying shrinkage, and microstructure. Results showed that the addition of GP reduced the fluidity and setting time of gangue powder—fly ash-base geopolymer (GPFAG), improved density, and decreased the water absorption rate of GPFAG. Moreover, its mechanical properties gradually improved. Compared with GPFAG0 (FAG with 0% GP), the 28-d compressive and flexural strengths of GPFAG50 (FAG with 50% GP) increased by 246.4% and 136.8%, respectively. The incorporation of GP increased the drying shrinkage. The results of XRD and FTIR analyses showed that the addition of GP increased the production of amorphous silica–aluminate gels, such as N-S-A-H and C-S-A-H. Moreover, strong Si-O-T vibrational peaks appeared in the range 743–1470 cm^−1^, characterizing the GPFAG strength and reaction degree.

## 1. Introduction

China is the largest consumer of coal in the world. Coal resource utilization generates large amounts of solid waste, such as fly ash (FA) and gangue. Solid waste is a critical factor affecting the sustainable development of society [1,2,3]. Devising a method for efficiently utilizing solid wastes to alleviate environmental pressure and realize the recycling of coal resources is a current research focus. In the late 1970s, a new silica–aluminate gel material with a three-dimensional reticular spatial structure ranging from amorphous to semi-crystalline was prepared by French scholar Joseph Davidovits using an alkaline solution and metakaolin, naming it ground geopolymer [4,5]. Less harmful gases, such as CO, CO_2_, and SO_2_, are generated during the production and preparation of geopolymers. Moreover, they exhibit high-quality characteristics such as early setting, early strength, high durability, and high fire resistance. Consequently, they have received considerable attention from local and international scholars.

Gangue is a by-product of coal mining and washing and mainly includes three major categories: digging gangue, coal selection gangue, and natural gangue. accounting for 10–20% of the total coal mixture [6,7,8]. The massive accumulation of gangue will cause dust, spontaneous combustion, etc., and the sulphide contained in it will pollute the atmosphere and water sources. This will cause further harm to the environment and, at the same time, pose a great danger to health [6]. In recent years, gangue has typically been used as concrete aggregate [9,10]. Some scholars have investigated and proposed the replacement of traditional coarse aggregate by crushed gangue to produce concrete [11,12,13,14,15]. They found that the strength of gangue concrete is approximately 20% lower than that of ordinary concrete [11,13]. The production of concrete consumes considerable amounts of energy and resources. Therefore, a greener and more environmentally friendly approach must be implemented to deal with gangue. The main components of gangue are Si and Al, which can be used as raw materials for geopolymer preparation. Moreover, compared with the production of cement-based materials, the preparation of geopolymers consumes less energy and generates fewer carbon emissions. In the field of construction, geopolymers exhibit desirable characteristics, such as high corrosion resistance and excellent mechanical properties and durability [16]. Accordingly, the use of geopolymer technology to treat gangue can realize gangue resource utilization. Moreover, with this technology, a high-performance green building material can be produced to replace traditional cement and reduce carbon production and energy consumption.

The internal crystal structure of gangue is considerably stable, and its organic carbon content is high. Consequently, the activity of gangue’s volcanic ash is extremely low; it must be activated before gangue can be utilized. The main techniques that can be employed for this purpose include mechanical, thermal, chemical, and microwave activation methods. However, the reactivity of gangue is difficult to stimulate completely [17,18,19]. Accordingly, the mechanical–thermal composite activation of gangue powder (GP) has been widely studied [20,21,22,23,24]. Ma et al. [22] prepared alkali-excited gangue–slag concrete after calcination at 700 °C and found that its durability was better than that of ordinary concrete. Beata et al. [25] found that geopolymer samples exhibited the best compressive and flexural strengths when the particle size range of gangue powder (GP) was below 200 μm. Mateusz et al. [26] Mateusz et al. found that geopolymer foams from gangue had stable mechanical properties at temperatures up to 600 °C by investigating the refractoriness of gangue-based polymer foam concrete. Qiu et al. [27] employed microwave activation at 600–700 °C to prepare Coal Gangue-based Green Cemented Filling Body (GPGCFB). They found that increasing the gangue substitution rate could fill the pores inside the concrete matrix and improve working performance. The activated gangue also had excellent volcanic ash and microaggregate effects. Frias [28] activated the volcanic ash of gangue using mechanical and thermal activation composites. Li [29] used a hydrothermal technique to calcine gangue with CaO to create a high-calcium bonding system, thereby improving gangue activity. Zhao et al. [30] found that the calcination temperature and holding time played decisive roles in the thermal activation of gangue. Moreover, the preparation of geopolymer using calcined gangue revealed that the internal hydration products of the matrix were mainly N-A-S-H gels. Frasson et al. [31] found that the strength of the geopolymer prepared by calcining the gangue at 700 °C was only approximately 21 MPa at 28 d, and the dry shrinkage performance was high. Guo et al. [32] found that the geopolymer prepared by calcining gangue at an appropriate temperature had improved microscopic pore structure, fewer harmful pores, and improved collective compactness, thus enhancing its mechanical properties. Dong et al. [33] prepared cement mortar by calcining the fine aggregate of gangue at different fineness values. Results showed that the mechanical properties of test blocks improved with decreasing gangue fineness. Considerable amounts of FA, a major solid waste by-product of coal-fired power plants, are a convenient and readily available source not only of aluminosilicate for geopolymers but also of aluminosilicate with relatively high reactivity in alkaline media. Studies on the use of geopolymer technology to treat gangue are limited. Similarly, investigations on the mechanism for the geopolymerization of gangue and FA as well as the effect of gangue on the performance of FA-based geopolymers (FAGs) are few.

In this study, the effect of GP addition on the mechanical properties of geopolymer was mainly investigated. Additionally, the effects of GP on the setting time, flowability, porosity, water absorption, and drying shrinkage of the geopolymer slurry were also investigated. X-ray diffraction (XRD) was used to investigate mineral phase changes during the reaction process. The functional groups in the reaction products were identified by Fourier transform infrared spectrometer (FTIR), which described the microscopic properties of the geopolymer, revealed the effect of GP on the properties of FAG, and provided a theoretical basis for the utilization of gangue as a civil engineering material.

## 2. Materials and Methods

### 2.1. Materials

**Figure 1:**
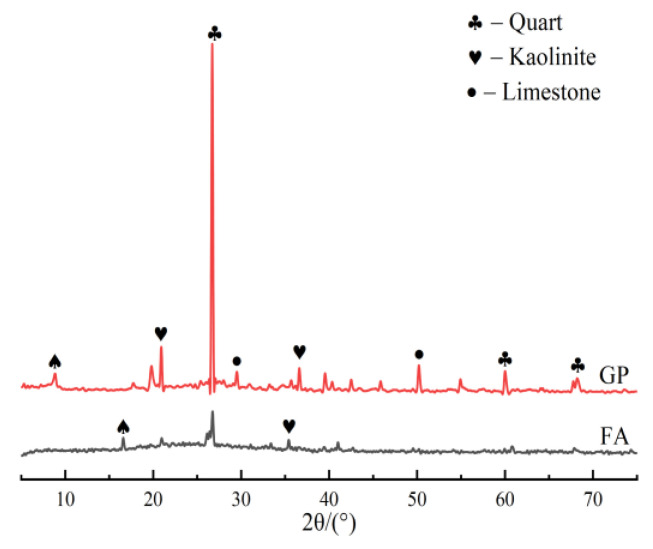
XRD analysis spectrum of GP and FA.

**Figure 2:**
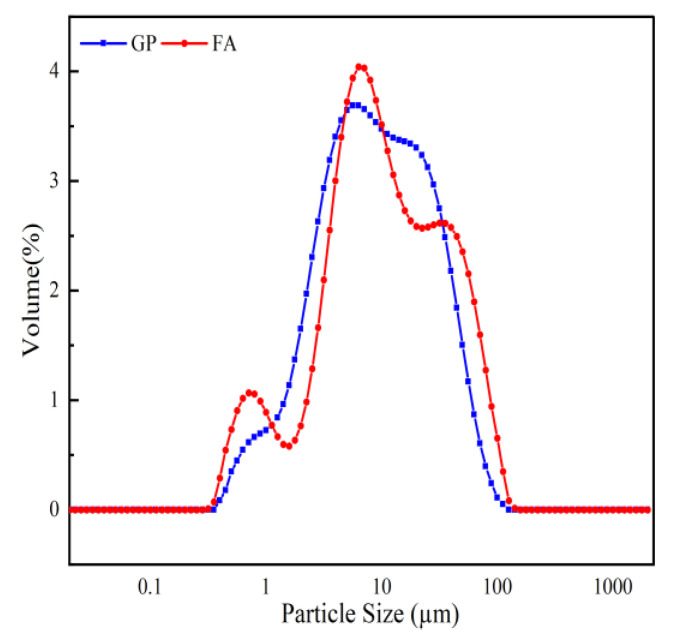
Particle size distribution.

The gangue used in this study was unburnt gangue obtained from the Ningxia region of China. It was crushed into small pieces by a jaw crusher and then screened to a particle size of less than 2.36 mm. The gangue was ground in a planetary ball mill at 300 rpm for 60 min. The apparent density of the calcined GP was 2575 kg/m^3^, and the fly ash (FA) was obtained from the class F fly ash produced by the Ningdong Yuanyang Lake Power Plant in Ningxia, China, with an apparent density of 2130 kg/m^3^; their XRD pattern characteristics are shown in Figure 1. The GP and FA crystalline phases are mainly quartz, kaolinite, and a small amount of calcite. And the compositions of GP and FA were all highly reactive Si, Al, and natural raw materials for geopolymer preparation. They react vigorously under alkaline conditions. This favors the development of geopolymer properties. The chemical compositions of GP and FA were determined by XRF, and the detailed results are listed in Table 1. Their particle size distributions are shown in Figure 2. Liquid waterglass has an initial modulus of 3.3; and NaOH with 98% purity and flake-like particles was used to adjust the modulus of the waterglass.

In this experiment, the base ratios used are as follows: liquid–solid ratio, 0.4; cement–sand ratio, 1:2.75; alkali exciter modulus, 1.4; and concentration, 27%. The percentage of GP used as FA replacement is 10–50% with a 10% gradient. To study the effect of GP on the performance of FAG, the specific ratios are listed in Table 2.

### 2.2. Specimens

To flake analytically pure NaOH, Na_2_SiO_3_ solution was first added and then stirred with a glass rod until NaOH was completely dissolved. Consequently, a waterglass solution with a modulus of 1.4 was prepared and subsequently used after resting for 24 h.

The geopolymer mortar was mixed using a JJ-5 cement mortar mixer produced by Wuxi Xiyi Building Material Instrument Factory. River sand, FA, and GP were poured into a mixing pot and slowly stirred for 1 min. An alkali exciter and water were poured into the pot and slowly stirred for 2 min. The slurry was rapidly stirred for 2 min and then poured into a 40 mm × 40 mm × 160 mm triplex mold. It was vibrated and pounded for 60 s, overlaid with film, placed in a standard maintenance room with a relative humidity of 95%, and maintained for 24 h. After demolding, it was transferred to the standard maintenance room until it reached a specified curing age before it was tested.

### 2.3. Test Procedure

**Figure 3:**
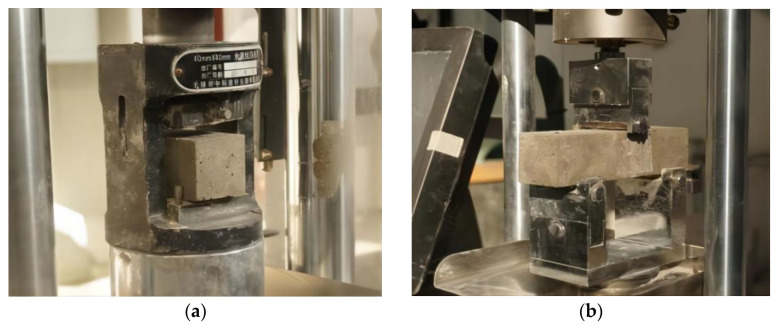
Universal press: (**a**) compressive strength test and (**b**) flexural strength test.

**Figure 4:**
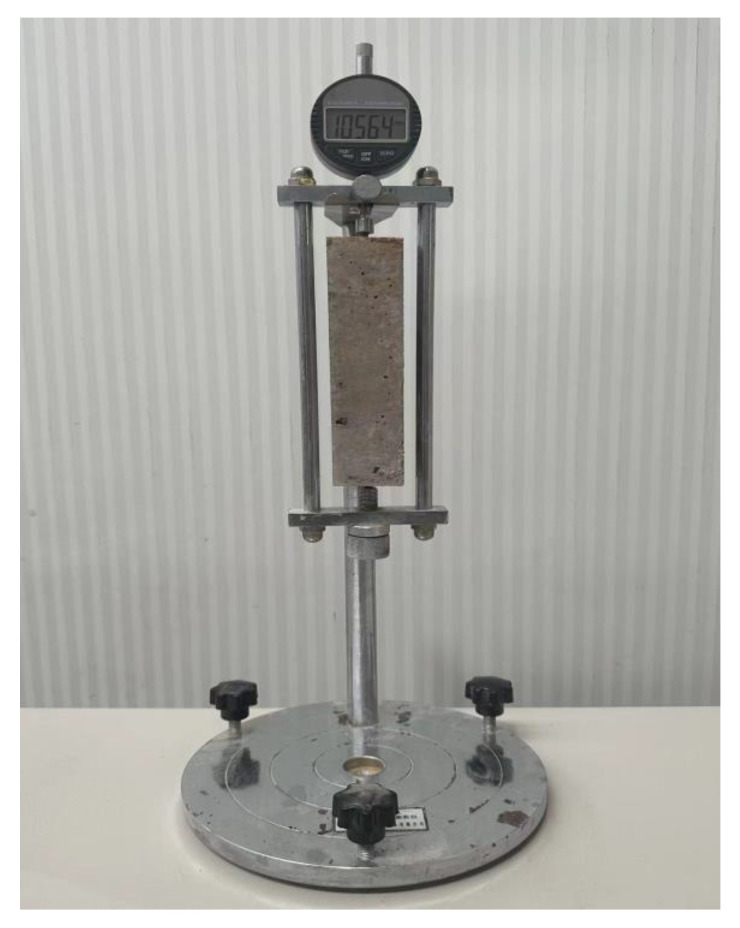
Vertical mortar shrinkage and expansion meter.

To determine the workability of GPFAG, the setting time was measured according to the method provided in the literature [34] with the help of the NJ-100S digital display mortar setting time tester produced by Hebei Blue Star Construction Instrument Co. (Langfang, China) The flowability of the freshly mixed mortar was determined by the jumping table method [35]. The maximum spreading diameter of the bottom surface of the paste and the diameter perpendicular to it were measured using calipers, respectively, and the average value of them was recorded as the flow value. The porosity and water absorption of the test blocks were also calculated by using Equations (1) and (2) provided in the standard ASTMC 642-13 [36]. Using a YAW-300F universal press (Figure 3) and the cement mortar strength test method (an ISO method) [37], with three specimens in each group, the loading rates were 2.4 kN/s and 50 N/s, respectively. Using an SP-175 vertical mortar shrinkage and expansion instrument (Figure 4), the drying shrinkage of FAG at 0, 1, 3, 5, 7, 14, 28, 56, and 90 d was measured according to the relevant provisions of JGJ/T70-2009 [34]:

(1) $$\mathrm{Water}\;\mathrm{absorption}=\frac{B\;-\;A}{A}\;\times \;100\%\;$$

(2) $$\mathrm{Porosity}=\frac{C\;-\;A}{C\;-\;D}\;\times \;100\%\;$$

where A, dry mass after drying at 110 ± 5 °C for 24 h; B, saturated mass after boiling for 5 h and cooling naturally for 5 h; and D, apparent mass of the wire suspended in water.

To avoid systematic errors in characterizing the samples (prepared by cutting or crushing) with potential microcracks formed under stress, the cores of samples that cracked after compressive strength tests were used. These cores were immersed in anhydrous ethanol for 3 d, followed by vacuum drying at 60 °C. The dried specimens were ground and passed through a 200 mesh sieve for XRD phase testing using an X’Pert powder X-ray diffractometer; the scanning range (2θ) was 5–75° in 0.02° steps. The molecular structure and chemical bonding of the samples were also investigated using a Nicolet 5DXC Fourier Infrared Spectrometer (FTIR) (Thermo Scientific, Waltham, MA, USA) with a scanning wavelength in the range of 400–4000 cm^−1^.

## 3. Results and Analyses

### 3.1. Setting Time

**Figure 5:**
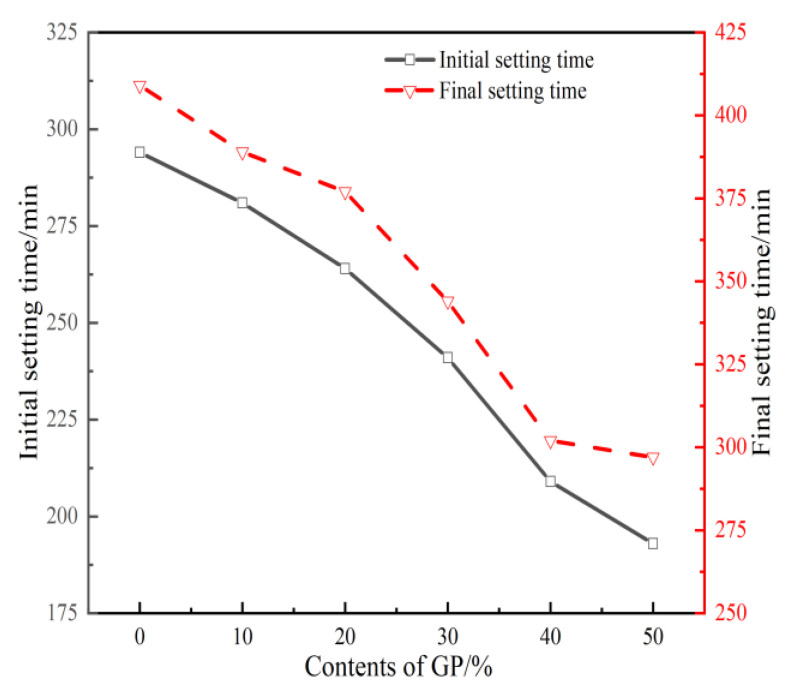
Setting time of GPFAG with different GP contents.

The effect of GP on the setting time of GPFAG is shown in Figure 5. The figure shows that with the incorporation of GP, the setting time of GPFAG is significantly reduced. The initial and final setting times of FAG are 294 and 409 min, respectively. Low-calcium FA has extremely low activity; consequently, its hydration level under alkaline conditions is also low. Moreover, the low Ca^2+^ content leads to the inability to form the C-(A)-S-H gel required for the rapid setting of GPFAG; consequently, the setting time is extremely long. When the amount of added calcined GP reaches 50%, the initial and final setting times of GPFAG50 are reduced to 193 and 297 min, respectively; these are 34.4% and 27.4% shorter than those of FAG, respectively. This indicates that the added GP accelerates the reaction rate of FAG. The coagulation time of a geopolymer mainly depends on the amounts of alkali exciter and active Si^4+^ and Al^3+^ in the raw material [38,39]. In this study, the amount and concentration of the alkali exciter were constant in all groups. When the GP admixture is increased, the active Si content of GPFAG gradually increases, and the SiO_2_–Al_2_O_3_ molar ratio in the formed matrix increases. This leads to an increase in the reaction level of the slurry at an early stage. Eventually, the setting time of the GPFAG will be significantly reduced.

### 3.2. Fluidity

**Figure 6:**
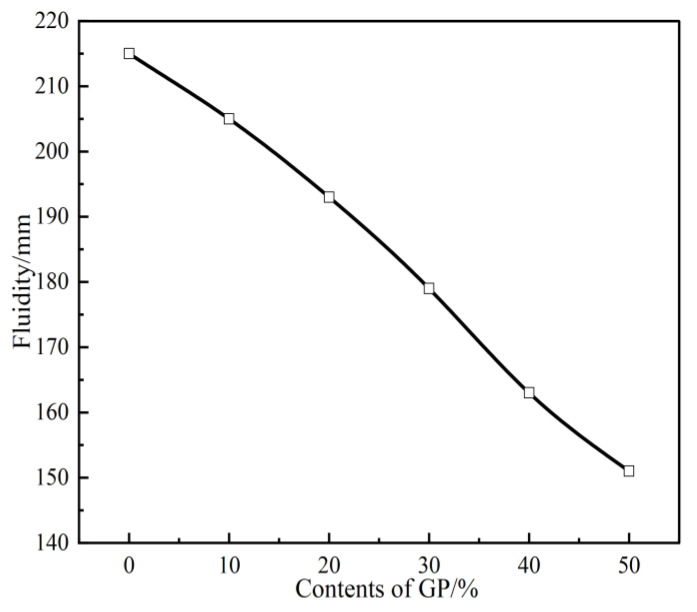
Fluidity of GPFAG with different GP contents.

Figure 6 shows the effect of different GP contents on the fluidity of FAG. The figure shows that the fluidity of GPFAG0 is 215 mm. With increasing GP content, the degree of flow of GPFAG decreases. With the addition of GP, the fluidity values of GPFAG in the experimental range are 205, 193, 179, 163, and 151 mm; these are 4.7%, 10.2%, 16.7%, 24.2%, and 29.8% lower than those of GPFAG0, respectively. The change in the fluidity of GPFAG is mainly attributed to the variations in the physical properties of raw materials. After fine grinding, the GP particles become smaller; this increases the specific surface area, which exceeds that of FA. Gangue has a high burn loss, and the GP after calcination at 600 °C has strong water adsorption [32,40]. The water absorption rate of the calcined GP can reach 32.4%, which is much higher than that of the FA (22.1%). The synergistic effect of various factors causes the GPFAG to consume more water during the mixing process. Finally, the fluidity of GPFAG is reduced.

### 3.3. Porosity

**Figure 7:**
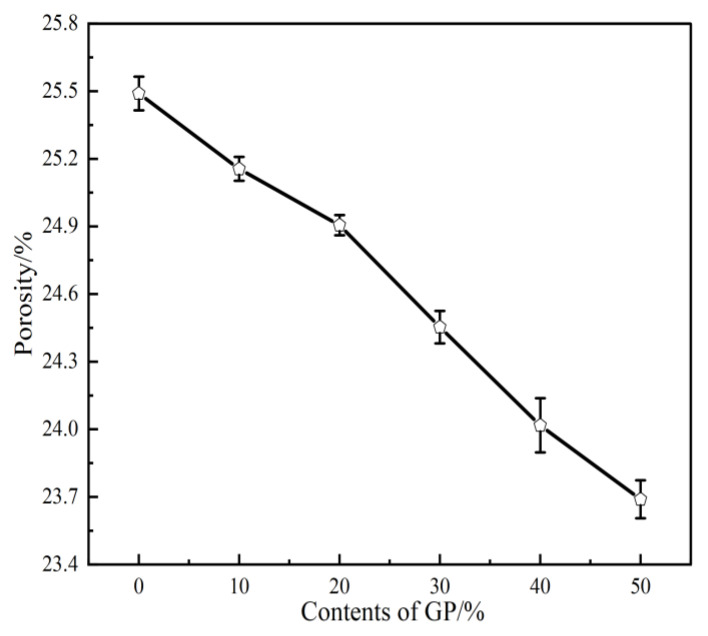
Porosity of GPFAG with different GP contents.

As shown in Figure 7, the effect of various GP contents on the porosity of FAG is tested. The figure indicates that the porosity of FAG decreases with increasing GP content. The porosity of the test block is 25.5% when GP is not incorporated. The porosity values of GPFAG under the experimental conditions are 25.2%, 24.9%, 24.5%, 24.0%, and 23.7%, which are 0.3%, 0.6%, 1.0%, 1.5%, and 1.8% lower than the porosity of GPFAG0, respectively. This is because calcined GP has high activity and a considerable volcanic ash effect. With the incorporation of GP, the level of depolymerization–condensation reaction inside the geopolymer increases, and the silica-aluminate cementitious materials generated, such as N-A-S-H, C-S-(A)-H, etc., precipitated rapidly inside the test blocks and filled the pores to a certain extent [27,30]. Moreover, the apparent density of GP is higher than that of FA, which leads to the rapid precipitation of substances generated by the reaction inside the matrix along with unreacted GP to fill the pores. Consequently, compactness is enhanced, and porosity is reduced [40].

### 3.4. Water Absorption

**Figure 8:**
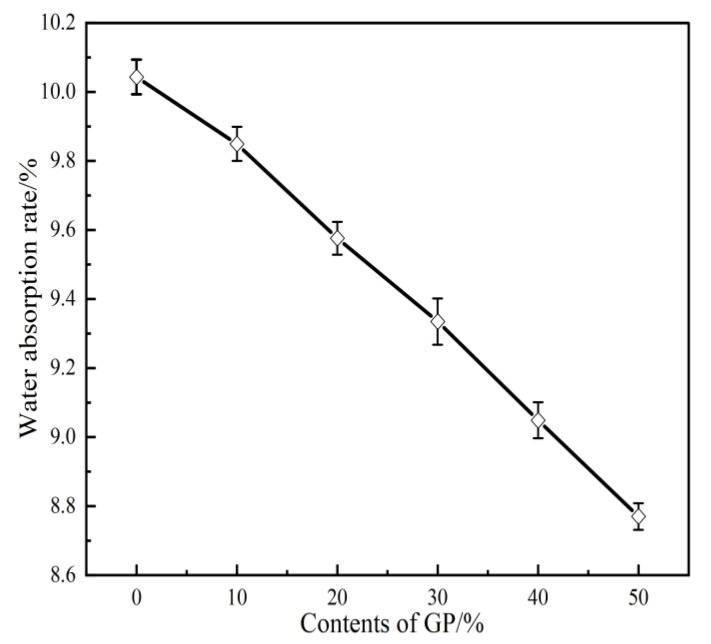
Water absorption rate of GPFAG with different GP contents.

Figure 8 depicts the effect of different GP contents on the water absorption rate of the GPFAG. The water absorption rate of the geopolymer is observed to decrease gradually with an increasing GP substitution rate. When GP is not incorporated (GPFAG0), the water absorption rate reaches 10%. When GP, whose activity is higher than that of FA, is activated, the depolymerization–condensation reaction inside the test block accelerates. Moreover, the amount of product generated increases and fills the internal pores of the test block. This causes the internal structure of the matrix to become more compact, preventing the entry of a considerable amount of external water into the matrix [28]; eventually, porosity decreases. Under the experimental conditions, with the incorporation of 10–50% GP (at 10% increments), the water absorption rate of the matrix decreases by 0.2%, 0.4%, 0.7%, 1.0%, and 1.2%, respectively, compared with that of GPFAG0.

### 3.5. Compressive Strength

**Figure 9:**
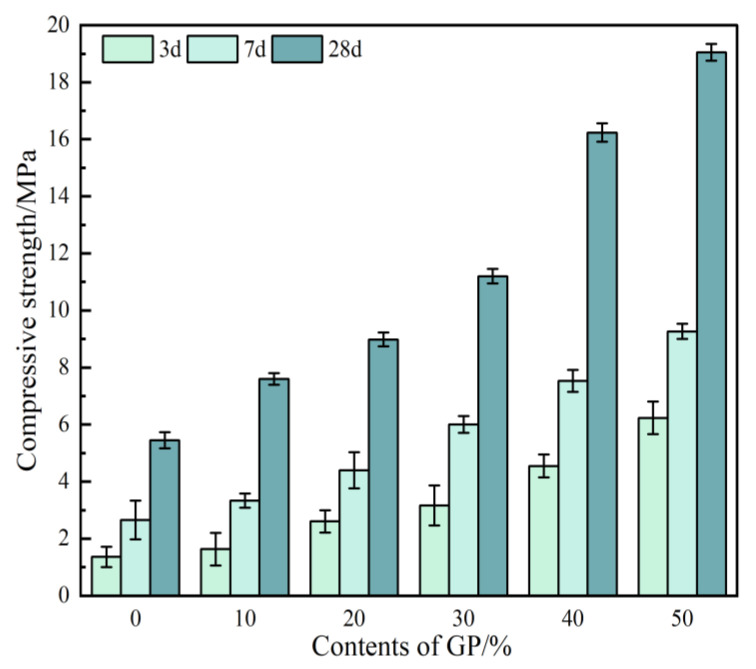
Compressive strength of GPFAG with different GP contents.

Figure 9 depicts a schematic of the compressive strength of the GPFGA versus different GP contents. The figure indicates that under the same conditions, the compressive strength increases with the GP content. The compressive strengths of GPFAG0 at 3, 7, and 28 d are 1.4, 2.7, and 5.5 MPa, respectively. When the GP content reaches 50% (GPFAG50), the compressive strengths at 3, 7, and 28 d are 6.2, 9.3, and 19.1 MPa, respectively; these are 345.2%, 243.2%, and 246.4% higher than those of GPFAG0, respectively. The foregoing indicates that the incorporation of GP substantially improves the compressive strength of GPFAG. The addition of GP can promote the formation of numerous composite structures. These structures improve the stability of the internal structure of the GPFAG while forming a dense ground geopolymer matrix. The greater the GP content, the more distinct the phenomenon. In addition, after the calcination of gangue, volcanic ash activity increases significantly. A considerable amount of GP admixture improves the level of depolymerization–condensation reaction inside the FAG matrix, generating a considerable amount of amorphous N-A-S-H and C-S-(A)-H gels. When subjected to external load, these generated gels play a certain role in supporting and protecting the interior of the matrix [32], thus improving the compressive strength of GPFAG, which is more similar to the results in the literature [31].

### 3.6. Flexural Strength

**Figure 10:**
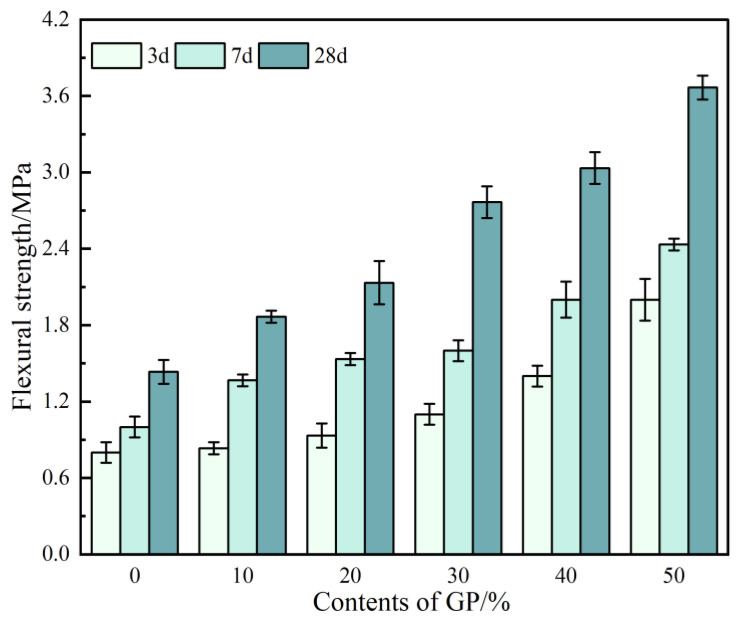
Flexural strength of GPFAG with different GP contents.

The effects of different GP contents on the flexural strength of GPFAG are shown in Figure 10. Similar to the effect of GP on compressive strength, the GP admixture has a positive effect on the flexural strength of GPFAG; that is, the flexural strength of GPFAG increases with the GP content. The flexural strengths of GPFAG0 at 3, 7, and 28 d are 0.8, 1.0, and 1.4 MPa, respectively. When the GP content is 50% (GPFAG50), the flexural strengths at 3, 7, and 28 d increase to 2.0, 2.4, and 3.7 MPa, which are 150%, 143.3%, and 136.8% higher than those of GPFAG0, respectively. The flexural strength of a geopolymer is related to its pore space [41,42]. The GP particles became smaller through fine grinding and calcination, improving the microaggregate effect. After the incorporation of GP, the pore structure inside the GPFAG matrix improves due to the microaggregate effect. This enhances the internal density, consequently improving the flexural strength of GPFAG.

### 3.7. Drying Shrinkage of Geopolymer Specimens

**Figure 11:**
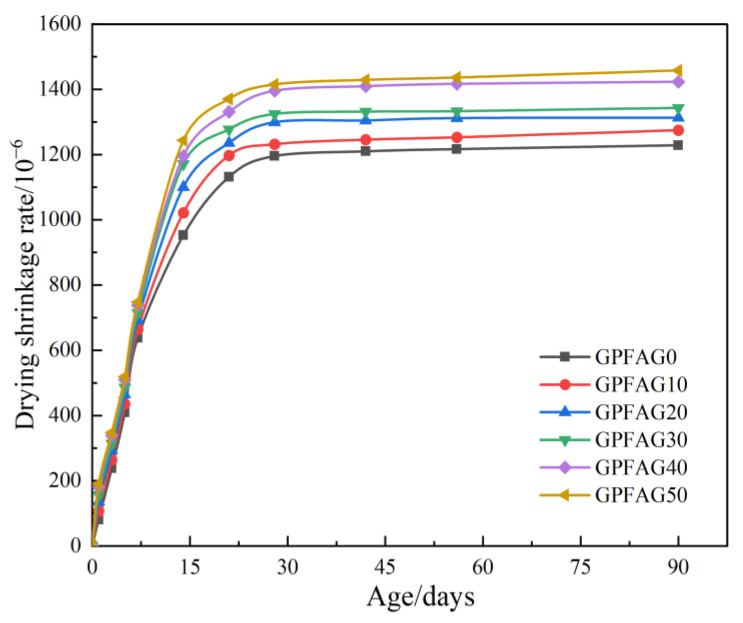
Drying shrinkage of GPFAG with different GP contents.

The effect of GP on the drying and shrinkage properties of the geopolymer is shown in Figure 11. The shrinkage rate of GPFAG gradually accelerates during the test period, particularly from 0 to 14 d, and then decelerates from 14 to 28 d. The shrinkage rate of GPFAG on day 28 can reach 1228.1 × 10^−6^. The incorporation of GP evidently increased the drying shrinkage rate. On day 28, under experimental conditions, the shrinkage rates of GPFAG with different GP contents are 1274.3 × 10^−6^, 1312.9 × 10^−6^, 1342.9 × 10^−6^, 1422.8 × 10^−6^, and 1457.6 × 10^−6^, which are 3.76%, 6.91%, 9.35%, 13.9%, and 18.9% higher than those of GPFAG0, respectively. This is because during the internal hydration of GPFAG over time, water is gradually consumed by the reaction under dry conditions, and some of the water evaporates [43]. Moreover, water is the reaction medium for the internal depolymerization–condensation reaction in GPFAG. However, most of the gelling material in GPFAG and the resulting silica–aluminate gels do not chemically bond with water [44]. The water in the matrix gradually evaporates under drying conditions as the internal depolymerization–condensation reaction continues. This causes the water demand and water loss inside the matrix to increase simultaneously, resulting in the drying shrinkage of GPFAG.

### 3.8. XRD Analysis

**Figure 12:**
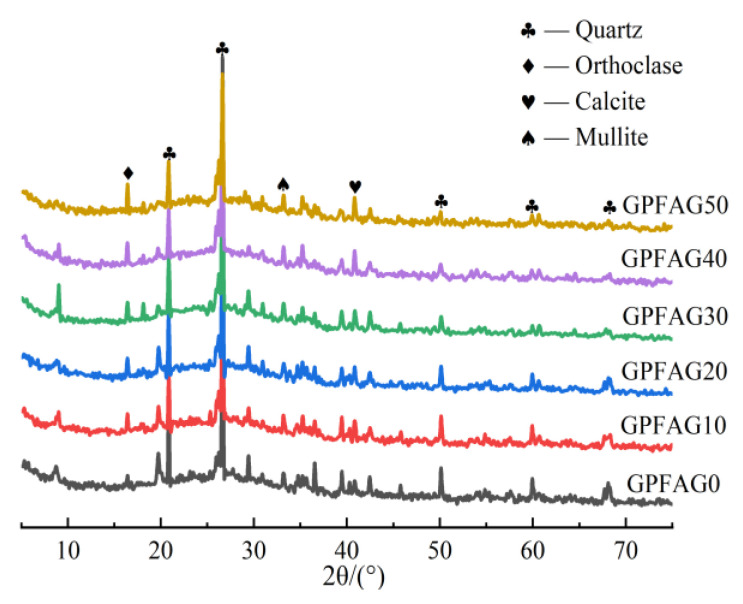
XRD pattern of GPFAG with different GP contents.

The XRD patterns of the GPFAG with different GP concentrations after 28 d of curing are shown in Figure 12. The figure indicates that the GP admixture has a considerable effect on the characteristic diffraction peaks of GPFAG. The main composition of GPFAG0 is a quartz phase with a small amount of silica–aluminate gel. The incorporation of GP does not generate a new crystalline phase inside the FAG matrix. Additionally, the diffraction peaks of the quartz phase inside the matrix gradually decrease, whereas those of the silica–aluminate gel phase gradually increase. This indicates that the addition of GP improves the reaction level inside the matrix. The unreacted GP and FA particles can be firmly embedded in and wrapped by the microstructure, thus improving the mechanical properties of GPFAG. Further, the existence of wide diffraction peak packets in the range 15–40° indicates that the hydration products of the geopolymer are mainly amorphous silica–aluminate gels [45,46], such as N-A-S-H, C-A-S-H, and C-S-H, which are mainly related to the high reactivity of GP.

### 3.9. FTIR Analysis

**Figure 13:**
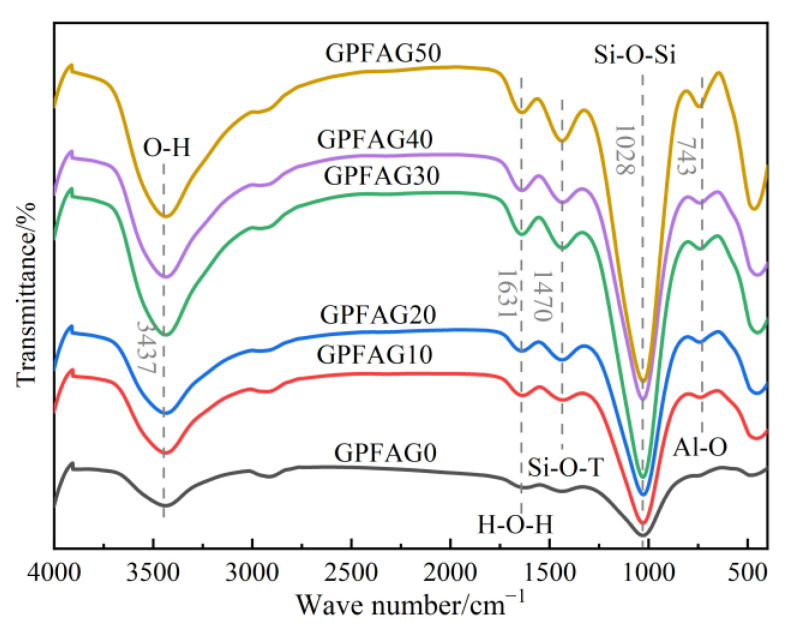
FTIR spectra of GPFAG with different GP contents.

The phase transition (molecular and bond structures) of the GPFAG is detected based on FTIR absorption peaks. Figure 13 shows the FTIR analysis of the GPFAG samples containing different amounts of calcined GP at 28 d. A significant hydroxyl (–OH) vibrational peak occurs at 3437 cm^−1^, and a –OH bending vibrational peak is observed at 1637 cm^−1^, characterizing the chemically bound water produced during geopolymerization [47]. The unreacted FA and small amount of GP, as well as the reaction products in the composition, are SiO_4_ and AlO_4_ tetrahedral structures. The strong absorption bands from 743 to 1470 cm^−1^ correspond to the asymmetric stretching vibration peaks of the Si-O-Si and Al-O-Si bonds. These indicate the occurrence of depolymerization–condensation reactions and the generation of more amorphous silica–aluminate gels. The N-A-S-H and C-(A)-S-H gels potentially compete with the crystalline phases in the unreacted raw material, resulting in a higher intensity of the vibrational peaks with increasing GP doping. This can indicate the formation of a continuous mesh of [Si] O-FAG [48,49]. As shown in the figure, the intensity of the absorption band at 1028 cm^−1^ increases with GP doping, indicating an increasing degree of depolymerization–condensation reaction. This is typically related to the formation of N-A-S-H and C-(A)-S-H gel phases inside the geopolymer [50]. The presence of Al-O near the 743 cm^−1^ characteristic peak indicates the precipitation of [AlO_6_] in the matrix [51]. This precipitation is enhanced with increasing GP content, showing that the depolymerization–condensation reaction is more adequate at this time. This is the reason for the subsequent increase in the strength of GPFAG with increasing GP content.

## 4. Conclusions

(1) The setting time of GPFAG can be improved by adding GP; it significantly decreases with increasing GP content. Influenced by the liquid–solid ratio, the addition of 50% GP (i.e., GPFAG50) reduces the fluidity, porosity, and water absorption rates by 29.8%, 1.8%, and 1.2%, respectively, compared with those of GPFAG0.
(2) The incorporation of GP improves the mechanical properties of GPFAG, and strength increases with the GP content. The 28-d flexural and compressive strengths of GPFAG with 10–50% (at 10% intervals) GP content compared with those of GPFAG0 increase by 136.8% and 246.4%, respectively.
(3) Based on the drying shrinkage results, the addition of GP induces an increase in the drying shrinkage of FAG due to a simultaneous increase in water demand and water loss within the matrix under drying conditions.
(4) The XRD and FTIR analyses show that the incorporation of GP reduces the amount of unreacted quartz phase inside the matrix. The addition of GP substantially enhances the depolymerization–condensation reaction inside the matrix. Moreover, GP doping generates a considerable amount of amorphous silica–aluminate gels, which contain substantial amounts of Si-O-Si, Si-O-Al and chemically bound water. This contributes to the formation of a dense, continuous, three-dimensional network structure, which enhances the strength of GPFAG.

## Figures and Tables

**Table 1:** Chemical composition (%).

Materials	Al_2_O_3_	SiO_2_	Fe_2_O_3_	CaO	Na_2_O	K_2_O	SO_3_
FA	26.38	47.85	8.43	5.81	1.11	2.22	1.94
GP	25.1	58.5	5.81	4.25	0.551	2.56	0.445

**Table 2:** Calcined gangue–FAG ratio (kg/m^3^).

Code	FA	GP	Sand	Alkali Exciter		Water
				Na_2_SiO_3_	NaOH	
GPFAG0	480	0	1320	164.24	27.76	119.9
GPFAG10	432	48				
GPFAG20	384	96				
GPFAG30	336	144				
GPFAG40	288	192				
GPFAG50	240	240				

## Data Availability

Not applicable.

## References

[1] Wang Q. (2013). China’s citizens must act to save their environment. Nature.

[2] Yuan H.J. (2018). The future of coal in China. Resour. Conserv. Recycl..

[3] Wang Q., Li R.R. (2016). Journey to burning half of global coal: Trajectory and drivers of China’s coal use. Renew. Sustain. Energy Rev..

[4] Davidovits J. (1991). Geopolymer: Inorganic polymeric new materials. J. Therm. Anal. Calorim..

[5] Davidovits J. (1976). Process for the Fabrication of Sintered Panels and Panels Resulting from the Application of This Process. U.S. Patent.

[6] Zhang Y., Ling T.C. (2020). Reactivity activation of waste coal gangue and its impact on the properties of cement-based materials—A review. Constr. Build. Mater..

[7] Magdalena M.K., Fabiańska M.J. (2011). Application of organic petrology and geochemistry to coal waste studies. Int. J. Coal Geol..

[8] Laura C.M., Medina C., Rojas I.M.S., Frías M. (2019). Water transport in binary eco-cements containing coal mining waste. Cem. Concr. Compos..

[9] Chen P., Zhang L., Wang Y., Fang Y., Zhang F., Xu Y. (2021). Environmentally friendly utilization of coal gangue as aggregates for shotcrete used in the construction of coal mine tunnel. Case Stud. Constr. Mater..

[10] Frías M., Rojas I.M.S., García R., Valdés A.J., Medina C. (2012). Effect of activated coal mining wastes on the properties of blended cement. Cem. Concr. Compos..

[11] Gruber K.A., Ramlochan T., Boddy A., Hooton R.D., Thomas M.D.A. (2001). Increasing concrete durability with high-reactivity metakaolin. Cem. Concr. Compos..

[12] Moghadam M.J., Ajalloeian R., Hajiannia A. (2019). Preparation and application of alkali-activated materials based on waste glass and coal gangue: A review. Constr. Build. Mater..

[13] Koshy N., Dondrob K., Hu L.M., Wen Q.B., Meegoda J.N. (2019). Synthesis and characterization of geopolymers derived from coal gangue, fly ash and red mud. Constr. Build. Mater..

[14] Zhang Y., Wang Q., Zhou M., Fang Y., Zhang Z. (2020). Mechanical properties of concrete with coarse spontaneous combustion gangue aggreagate (SCGA): Experimental investigation and prediction methodology. Constr. Build. Mater..

[15] Mahmood K., Dabbaghi F., Sadeghi-Nik A., Dehestani M. (2020). Mechanical performance of green concrete produced with untreated coal waste aggregates. Constr. Build. Mater..

[16] García-Giménez R., Frías M., Arribas I., Vegas I., de la Villa R.V., Rubio V. (2018). Freeze-thaw effect on the durability of binary cements containing activated coal-mining waste. Constr. Build. Mater..

[17] Mejia-Ballesteros J.E., Rodier L., Filomeno R., Savastano Jr H., Fiorelli J., Rojas M.F. (2023). Effect of activated coal waste and treated Pinus fibers on the physico-mechanical properties and durability of fibercement composites. Constr. Build. Mater..

[18] Seiffarth T., Hohmann M., Posern K., Kaps C. (2013). Effect of thermal pre-treatment conditions of common clays on the performance of clay-based geopolymeric binders. Appl. Clay Sci..

[19] Sedira N., Castro-Gomes J., Kastiukas G., Zhou X., Vargas A. (2017). A review on mineral waste for chemical-activated binders: Mineralogical and chemical characteristics. Min. Sci..

[20] Duxson P., Fernández-Jiménez A., Provis J.L., Lukey G.C., Palomo A., van Deventer J.S.J. (2007). Geopolymer technology: The current state of the art. J. Mater. Sci..

[21] Dellisanti F., Valdrè G. (2012). The role of microstrain on the thermostructural behaviour of industrial kaolin deformed by ball milling at low mechanical load. Int. J. Miner. Process..

[22] Ma H., Zhu C., Wu H., Chen J., Sun J., Liu J. (2020). Study on compressive strength and durability of alkali-activated coal gangue-slag concrete and its mechanism. Powder Technol..

[23] Liu Y., Ling C.T., Wang M., Wu Y.Y. (2021). Synergic performance of low-kaolinite calcined coal gangue blended with limestone in cement mortars. Constr. Build. Mater..

[24] Al-Akhras N.M. (2006). Durability of metakaolin concrete to sulfate attack. Cem. Concr. Res..

[25] Figila B., Korniejenko K., Bulut A., Şahin B., Azizağaoğlu G., Plawecka K., Kozub B. (2023). Influence of the Size of Milled Coal Gangue Particles on the Mechanical Properties of Geopolymers. Mater. Proc..

[26] Sitarz M., Figiela B., Lach M., Korniejenko K., Mróz K., Castro-Gomes J., Izabela H. (2022). Mechanical Response of Geopolymer Foams to Heating—Managing Coal Gangue in Fire-Resistant Materials Technology. Energies.

[27] Qiu J.S., Cheng K., Zhang R.Y., Gao Y., Guan X. (2022). Study on the influence mechanism of activated coal gangue powder on the properties of filling body. Constr. Buuld. Master..

[28] Frías M., Villa R., Rojas M., Medina C., Valdes A., Jantzen C. (2012). Scientific Aspects of Kaolinite Based Coal Mining Wastes in Pozzolan/Ca(OH)_2_ System. J. Am. Ceram. Soc..

[29] Li C., Wan J.H., Sun H.H., Li L.T. (2010). Investigation on the activation of coal gangue bya new compound method. J. Hazard. Mater..

[30] Zhao Y.B., Yang C.Q., Li K.F., Qu F., Yan C.Y., Wu Z.R. (2022). Towardunderstanding the activation and hydration mechanisms of composite activated coal gangue geopolymer. Constr. Build. Mater..

[31] Frasson B.J., Rocha J.C. (2023). Drying shrinkage behavior of geopolymer mortar based on kaolinitic coal gangue. Case. Stud. Constr..

[32] Guo Z.H., Xu J.J., Xu Z.H., Gao J.M., Zhu X.L. (2022). Performance of cement-based materials containing calcined coal gangue with different calcination regimes. J. Build. Eng..

[33] Dong Z.C., Xia J.W., Fan C., Cao J.C. (2015). Activity of calcined coal gangue fine aggregate and its effect on the mechanical behavior of cement mortar. Constr. Build. Mater..

[34] (2009). Construction Mortar Basic Performance Test METHOD Standards.

[35] (2005). Test Method for Fluidity of Cement Mortar.

[36] (2022). Standard Test Method For Density, Absorption, and Voids in Hardened Concrete.

[37] (1999). Method of Testing Cements—Determination of Strength.

[38] Li X., Bai C., Qiao Y., Wang X., Yang K., Colombo P. (2022). Preparation, properties and applications of fly ash-based porous geopolymers: A review. J. Clean. Prod..

[39] Zhang L.Y., Ahmari S., Zhang J.H. (2011). Synthesis and characterization of fly ash modified mine tailings-based geopolymers. Constr. Build. Mater..

[40] Ji X.M., Ji D.P., Yang Z.X., Wang G.L., Huang X.F., Ma S.H., Li W.F. (2021). Study on the phase composition and structure of hardened cement paste during heat treatment. Constr. Build. Mater..

[41] Han Q., Wang A.N., Zhang J. (2022). Research on the early fracture behavior of fly ash-based geopolymers modified by molybdenum tailings. J. Clean. Prod..

[42] Liew Y.M., Heah C.Y., Li L.Y., Jaya N.A., Abdullah M.M.A., Jin T.S., Hussin K. (2017). Formation of one-part-mixing geopolymers and geopolymer ceramics from geopolymer powder. Constr. Build. Mater..

[43] Trincal V., Multon S., Benavent V., Lahalle H., Balsamo B., Caron A., Bucher R., Caselles L.D., Cyr M. (2022). Shrinkage mitigation of metakaolin-based geopolymer activated by sodium silicate solution. Cem. Concr. Res..

[44] Popovics S. (1975). Verification of relationships between mechanical properties of concrete like materials. AJER.

[45] Ryu G.S., Lee Y.B., Koh K.T., Chung Y.S. (2013). The mechanical properties of fly ash-based geopolymer concrete with alkaline activators. Constr. Build. Mater..

[46] Ibrahim M., Johari A.M.M., Rahman M.K., Maslehuddin M. (2017). Effect of alkaline activators and binder content on the properties of natural pozzolan-based alkali activated concrete. Constr. Build. Mater..

[47] Cheng Y., Ma H.Q., Chen H.Y., Shi J.X., Shi J., Li Z.H., Yu M.K. (2018). Preparation and characterization of coal gangue geopolymers. Constr. Build. Mater..

[48] Boke N.D., Birch G.M., Nyale S., Petrik L.F. (2015). New synthesis method for the production of coal fly ash-based foamed geopolymers. Constr. Build. Mater..

[49] Luo Y., Klima K.M., Brouwers J.H.H., Yu Q. (2022). Effects of ladle slag on Class F flyash geopolymer: Reaction mechanism and high temperature behavior. Cem. Concr. Compos..

[50] Onutai S., Osugi T., Sone T. (2023). Alumino-Silicate Structural Formation during Alkali-Activation of Metakaolin: In-Situ and Ex-Situ ATR-FTIR Studies. Mater. Proc..

[51] Garcia-Lodeiro I., Palomo A., Fernandez-Jimenez A., Macphee D.E. (2011). Compatibility studies between N-A-S-H and C-A-S-H gels. Study in the ternary diagram Na_2_O-CaO-Al_2_O_3_-SiO_2_-H_2_O. Cem. Concr. Res..

